# Quality of life, infection control, and complication rates using a novel custom-made articulating hip spacer during two-stage revision for periprosthetic joint infection

**DOI:** 10.1007/s00402-021-04274-4

**Published:** 2021-12-01

**Authors:** Andre Lunz, Georg W. Omlor, Gunter Schmidt, Babak Moradi, Burkhard Lehner, Marcus R. Streit

**Affiliations:** 1 Clinic for Orthopedics and Trauma Surgery, Center for Orthopedics, Trauma Surgery and Spinal Cord Injury, Heidelberg University Hospital, Schlierbacher Landstrasse 200a, 69118 Heidelberg, Germany; 2 Clinic for Orthopedics and Traumatology, University Medical Center Schleswig-Holstein, Campus Kiel, Kiel, Germany; 3 ARCUS Sportklinik, Pforzheim, Germany

**Keywords:** Two-stage revision, Periprosthetic joint infection, Antibiotic-loaded cement spacer, Articulating spacer, Metal-on-cement, Quality of life

## Abstract

**Introduction:**

Two-stage revision remains the gold standard treatment for most chronically infected and complex total hip arthroplasty infections. To improve patient outcome and reduce complication rates, we have developed a novel custom-made articulating hip spacer technique and present our short-term results.

**Materials and methods:**

Between November 2017 and November 2019, 27 patients (mean age 70 years) underwent two-stage revision for periprosthetic joint infection of the hip using the articulating spacer design described here. We retrospectively analyzed spacer-related complications as well as rates for complication, infection control, and implant survivorship after final reimplantation. Furthermore, we prospectively collected patient-reported health-related quality of life (HRQoL) scores prior to spacer implantation, with the spacer and after reimplantation of the new prosthesis.

**Results:**

An additional round of spacer exchange was performed in two patients (8.3%), persistent wound discharge was the reason in both cases. We had one (4.2%) spacer-related mechanical complication, a dislocation that was treated with closed reduction. After reimplantation, infection control was achieved in 96% with an implant survivorship of 92% after a mean follow-up time of 19 (range 7–32, SD 7.2) months. While the scores for VR-12 MCS, VAS hip pain and patient-reported overall satisfaction significantly improved after first stage surgery, the scores for WOMAC, UCLA and VR-12 PCS significantly improved after second stage surgery.

**Conclusions:**

Our two-stage approach for periprosthetic joint infection shows high infection eradication and implant survivorship rates at short-term follow-up. Spacer-related complication rates were low, and we achieved high patient satisfaction rates and low pain levels already during the spacer period. To further simplify comparison between different spacer designs, we propose a new hip spacer classification system.

## Introduction

Total hip replacement is one of the most successful procedures in orthopedic surgery, and an exponential rise in numbers of hip arthroplasty procedures is noted in a further ageing population. Subsequently, even a relatively low rate of periprosthetic joint infections (PJI) of 0.5–2% constitutes a major concern and burden to healthcare systems [1–3]. In case of an acute PJI, when the biofilm is still immature, an attempt to retain implants by performing surgical debridement with exchange of modular parts (DAIR) and avoid major revision surgery is often conducted. Nevertheless, approximately 30% of these patients will subsequently require revision surgery with implant exchange [4–6]. In chronic PJIs, when certain requirements are given and patients are carefully selected, one-stage revision has recently shown good clinical results with numerous advantages for the patient and healthcare system [7–9]. However, two-stage revision remains the “gold standard” treatment for most chronically infected and complex total hip arthroplasty infections with successful eradication rates up to 90% [10–14]. Mobile spacers, which create an articulation between the femur and acetabulum, have shown to successfully overcome well known disadvantages like periarticular scarring, soft tissue contractures, limb shortening, bone loss, and low functional results of Girdlestone hips and static spacers [10, 15–17]. To minimize the risk of acetabular bone wear, dislocation, spacer fracture and persistent infection, we have developed a custom-made antibiotic-loaded articulating hip spacer with a standard femoral stem endoskeleton and a polyethylene-free metal-on-cement articulation [18–20] and present our preliminary results in the setting of chronic PJI. Our study hypothesis was that the use of our spacer design reduces complication rates while improving infection control and patient satisfaction. The aims of our study were therefore to investigate (1) spacer-related complications, (2) infection control, implant survivorship, and complication rates after final reimplantation and (3) patient-reported health-related quality of life (HRQoL) based on standardized patient-reported outcome measurements (PROMs) for three points in time: prior to explantation, prior to reimplantation with spacer in situ, and after reimplantation of the new prosthesis.

## Materials and methods

### Patient selection and study summary

Approval was obtained from the local Medical Ethics Committee (S-449/2020). To gain a homogenous patient cohort we excluded PJIs of megaprostheses and hemiarthroplasties. Patients were prospectively included in our institutional arthroplasty registry, and we retrospectively reviewed a consecutive series of 27 cases treated with the articulating hip spacer technique described here between November 2017 and November 2019. All included patients were diagnosed with chronic PJI according either to the Musculoskeletal Infection Society [21] or the International Consensus Meeting [22] and underwent a two-stage revision using our custom-made articulating hip spacer with a metal-on-cement articulation for the interim period. Both, stage one- and stage-two surgery were performed by specialized surgeons at our certified trans-regional joint arthroplasty center.

### Surgical technique, interim period, and postoperative course after reimplantation

**Fig. 1:**
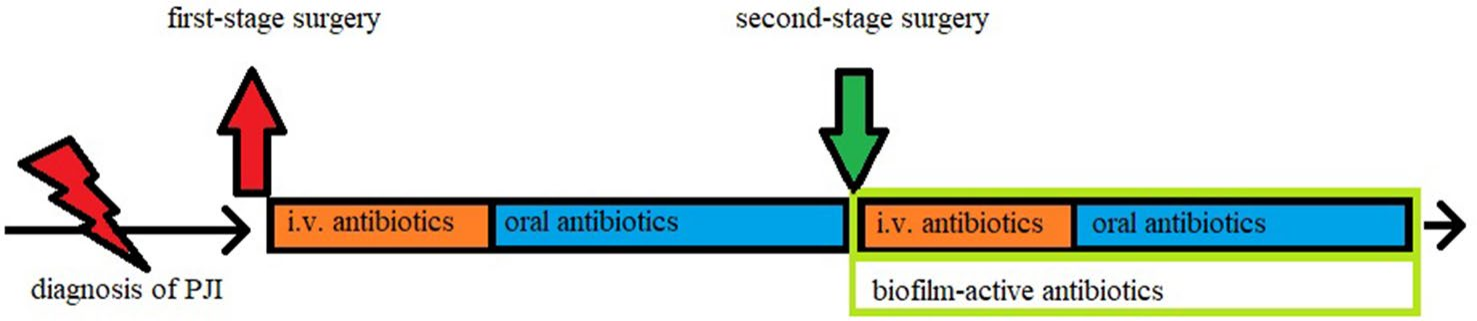
Our interdisciplinary approach for two-stage revision surgery in the setting of chronic PJI. Diagnosis was based on a variety of parameters, including clinical presentation, radiological work-up, laboratory workup, synovial analysis, and microbiological results after prolonged incubation of 2 weeks. Neither antibiotic holidays nor arthrocentesis were performed prior to reimplantation at second-stage surgery. Thorough debridement, the key element to overcome infection, was performed at both stages

**Fig. 2:**
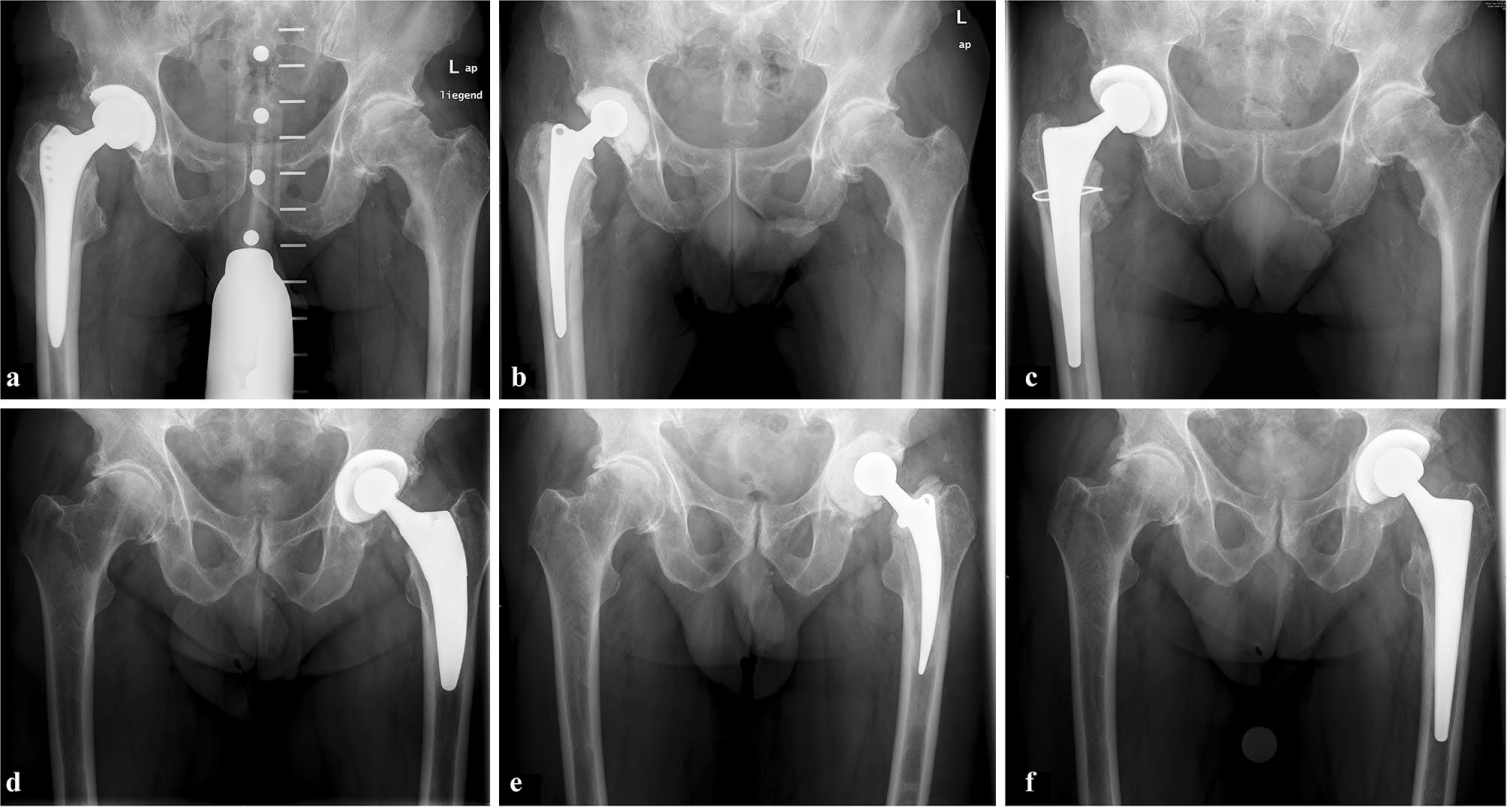
Representative radiographic images of two patients (**a**–**c** and **d**–**f**). From left to right, X-rays are provided of the same patient prior to explantation, with the articulating spacer in-situ, and after reimplantation of the new prosthesis. In both cases cement-free fixation was performed using a straight femoral stem (CLS Spotorno, Zimmer) and a press-fit acetabular cup (Allofit, Zimmer). If poor bone-stock was assumed intraoperatively a femoral cerclage (Cable-Ready, Zimmer) was used (**c**) before final implantation of the femoral stem to minimize the risk for an intraoperative femoral fracture

Our approach for two-stage revision in the setting of chronic or complex periprosthetic joint infections is described in Fig. 1. Representative radiographic images are provided in Fig. 2, showing two patients prior to explantation, with the spacer, and after reimplantation of the new prosthesis.

#### First-stage surgery

**Fig. 3:**
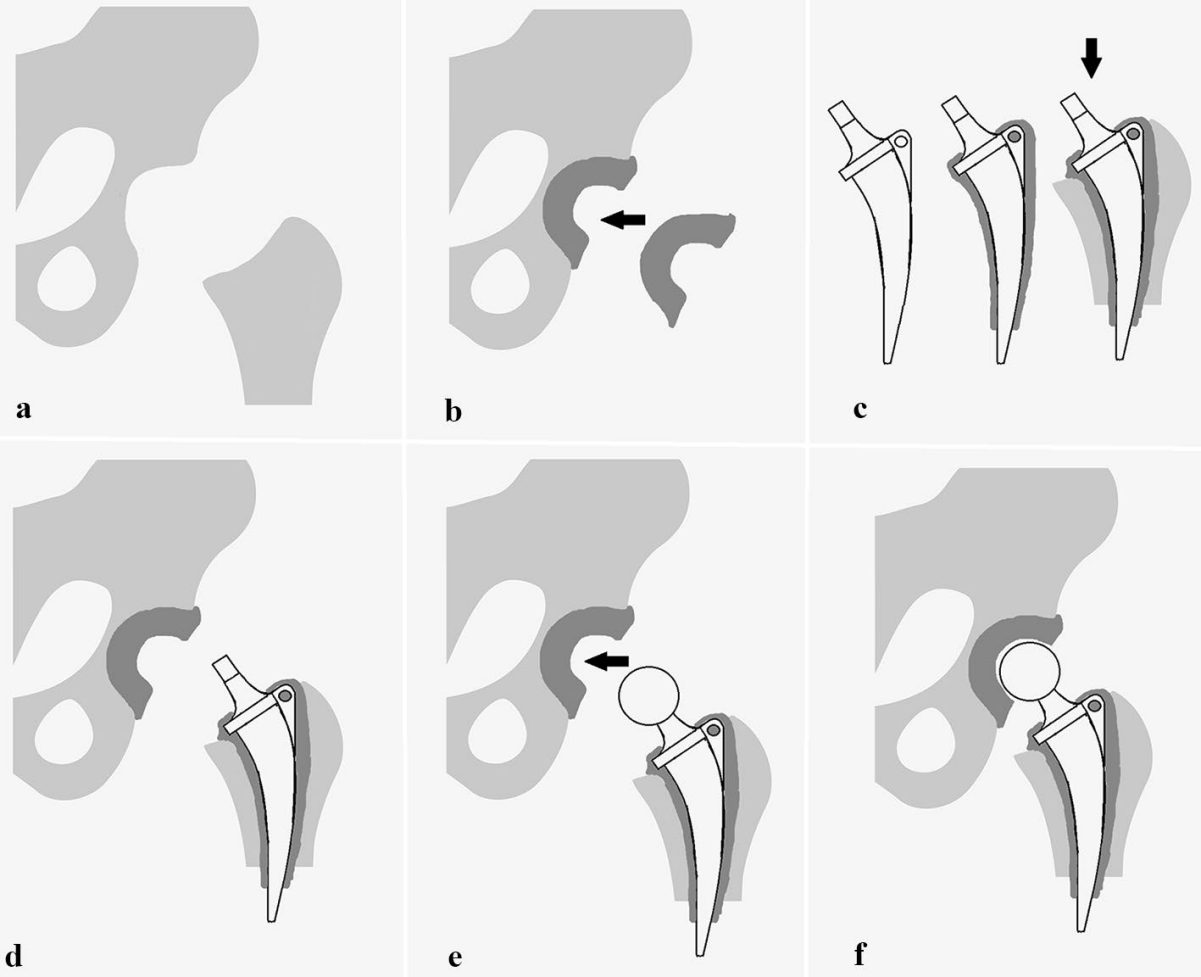
Illustration of our articulating hip spacer technique. **a** Situs after infected prosthesis is removed and radical debridement is performed. **b** Custom-made acetabular socket is formed from antibiotic-loaded bone cement and its implantation is performed. **c** Next, the femoral stem is wrapped with custom-made antibiotic-loaded bone cement and inserted into the proximal femur. To facilitate explantation at second-stage surgery, no cement is applied around the tip of the femoral stem. **d** After both spacer components (acetabular PMMA cup and PMMA-wrapped femoral stem) are in place, **e** the trial femoral head (size S-XL) is connected with the femoral stem, and a trial reduction maneuver is carefully performed. Clinical examination for stability, dislocation safety, and free range of motion is performed and the final femoral head size is chosen. **f** After final reduction, spacer implantation is completed

**Fig. 4:**
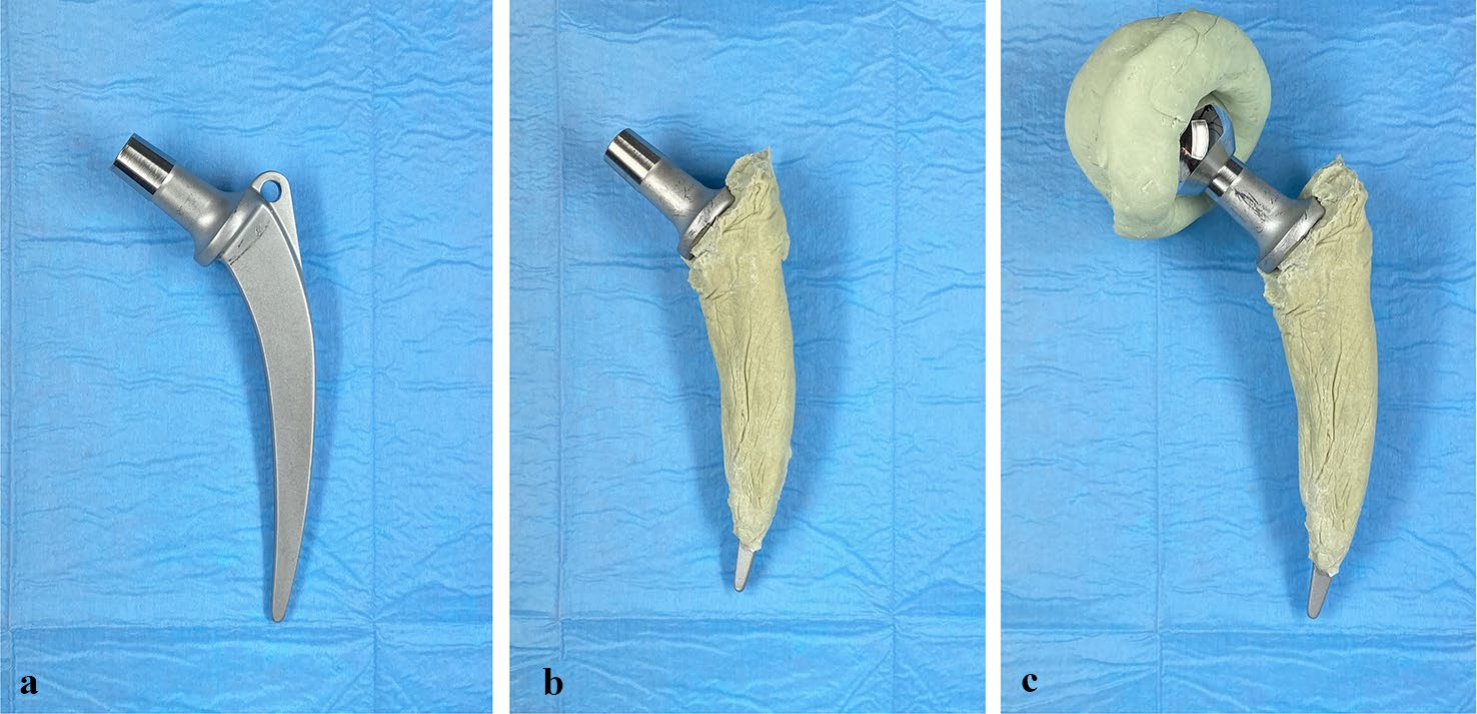
**a** We used an unsophisticated anatomically shaped femoral stem (Weber, Zimmer), **b** that was wrapped with antibiotic-loaded bone cement. To facilitate explantation at second-stage surgery the tip of the femoral stem was left free of any cement. **c** Our custom-med hip spacer consists of a hand-made acetabular cup formed out of antibiotic-loaded bone cement that is articulating with a metal femoral head (28–32 mm, S-XL, DePuy Synthes) connected with a femoral stem wrapped with antibiotic-loaded bone cement

All surgeries were performed through a modified direct lateral approach. First, diagnosis of a septic PJI is confirmed intraoperatively. Then, numerous tissue samples are taken for microbiological and histological examination while radical debridement and removal of all foreign material is performed. Then, the custom-made acetabular socket is formed by hand out of 40–80 g of antibiotic-loaded PMMA cement (Palacos R + G cement, Heraeus, Hanau, Germany). When possible, an additional antibiogram-specific antibiotic is added to the PMMA (3 g Vancomycin powder per 40 g of cement in most cases) and put as dough into the acetabular groove. Before PMMA polymerization has occurred an articulation groove is formed in the middle of the acetabular spacer using a plunger with a slightly larger head diameter (+ 4 mm) than the planned femoral head. To form a smooth groove, the plunger is continuously rotated and moved until PMMA polymerization has finished and the acetabular component is fixed. Cup orientation is aimed at an inclination angle of 45° and anteversion angle of 15°. Then the preparation of the femoral component is performed by wrapping the femoral stem (Weber Stem CS/CM/SM, Zimmer, Warsaw, IN, USA) in custom-made antibiotic-loaded cement (as described above). To facilitate explantation at second stage surgery, no cement is applied around the tip of the femoral stem. To avoid cement penetration into the bone for a strong fixation, the stem is continuously moved a few millimeters back and forwards until PMMA polymerization has completed. From our experience, this “deliberately loose cementation technique” provides sufficient stability while allowing easy removal without sacrificing bone stock during second-stage surgery. After both spacer components are in place, a metal head (28 mm- or 32 mm, S-2XL, DePuy Synthes, West Chester, PA, USA) is connected to the femoral stem, and reduction maneuver is carefully performed. An illustration of our spacer technique is given in Figs. 3 and 4.

First-stage surgery is finished with the insertion of a closed-suction drain (CSD) into the joint and closing the wound in common technique. Lastly retrograde application of 2 g vancomycin powder mixed with 2 g tranexamic acid is performed via the CSD.

### Interim period

All patients were advised to perform partial weight-bearing to prevent spacer-related mechanical complications during the interim period.

If the causative agent was known preoperatively, a targeted intravenous antibiotic therapy was started immediately after all samples were taken. Otherwise, empirical, broad-spectrum intravenous antibiotic therapy was administered and de-escalation to a targeted therapy was performed as soon as microbiological results were available. In all cases intravenous antibiotic therapy was continued for a total time of 7–14 days before being switched to an oral substance with good bone penetration.

In complex cases, with prolonged wound drainage or persistent signs of infection, a multi-stage procedure with an additional exchange of the spacer is sometimes required. Biofilm-active antibiotics, like rifampicin, were not used in the presence of a spacer [23]. In accordance with recent publications, antibiotic-free intervals or an arthrocentesis were not routinely performed prior to reimplantation [24–26]. Reimplantation was only planned if eradication of the infection was suggested by clinical parameters and laboratory results, most importantly a low to normal CRP-level.

### Second-stage surgery

All surgeries are performed using the same approach and care as described above (see chapter “first-stage surgery”). After extensive irrigation and final verification that all foreign, infected, sclerotic, or necrotic tissue has been removed, reimplantation of a new standard hip prosthesis (e.g., CLS Spotorno stem and Allofit acetabular cup (Zimmer Inc., Warsaw, IN, USA) is conducted. We generally prefer a cement-free press-fit fixation if the bone stock is adequate for cementless fixation.

### Postoperative course after reimplantation

Antibiotic therapy was performed as described above (see chapter “interim period”), with the exception that now the application of a biofilm-active drug such as rifampicin or ciprofloxacin is of upmost importance to protect the new prosthesis [23, 27, 28]. All patients were regularly monitored with laboratory and clinical examinations and discharged after wound healing has occurred and antibiotics were switched to an oral application.

### Outcome parameters

Our primary outcome measures were infection control, implant survivorship, and complication rates after reimplantation of the new prosthesis.

Our secondary outcome measures were patient-reported health-related quality of life (HRQoL) scores. We therefore collected and analyzed the following patient-reported outcome questionnaires (PROMs): Veterans RAND 12-Item Health Survey (VR-12) [29], Western Ontario and McMaster Universities Arthritis Index (WOMAC) [30], University of California at Los Angeles Activity Score (UCLA) [31], visual analog scale (VAS) for hip pain [32] and grading for patient satisfaction. All patients were asked to participate in the described PROMs at three different points in time: shortly prior to explantation, during the spacer period, and after final reimplantation.

### Statistical analysis

Descriptive statistics are presented as numbers of occurrence, percentage or arithmetic mean, and standard deviation (SD). Visualization was performed using boxplots and diagrams. The Friedman Test was used to compare the collected patient-reported outcome questionnaires prior to explantation, with the spacer, and after reimplantation. Kaplan–Meier survival analysis was used to estimate final implant revision rates. The level of significance was set at *p* < 0.05 for all statistical tests. The statistical analyses were performed using SPSS software (version 25.0; SPSS Inc, Chicago, IL, USA).

## Results

**Fig. 5:**
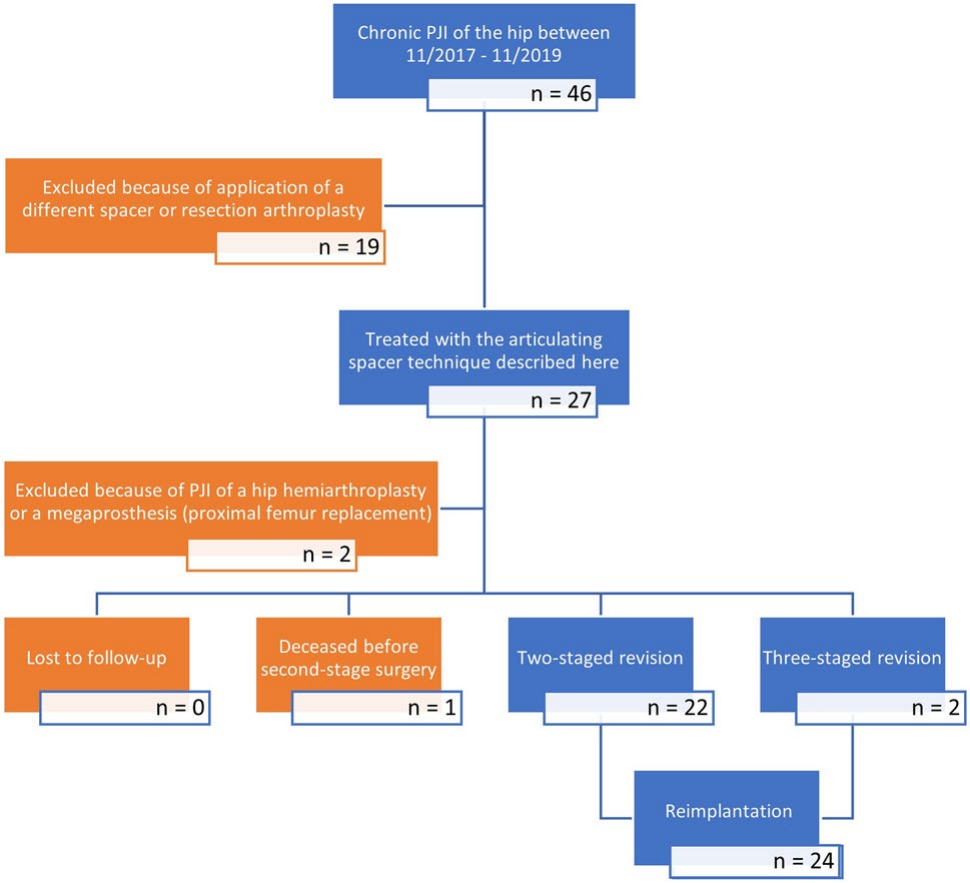
THAs treated for PJI in our department from 11/2017 to 11/2019

During the 2-year period (11/2017–11/2019), 27 patients were treated with our custom-made articulating hip-spacer technique in the setting of PJI of the hip. Of the 27 patients, 17 were men (63%) and a total of 3 patients (11%) had a prior history of revision surgery. One patient died before reimplantation from end-stage liver disease and was excluded from the analysis. Furthermore, two patients were excluded as they did not meet our inclusion criteria. One of them developed a PJI after proximal femur replacement (MUTARS megaprosthesis) in the setting of osteosarcoma of the proximal femur. He underwent successful two-stage revision without complications. The other one developed a PJI after implantation of hemiarthroplasty in the setting of traumatic femoral neck fracture. This patient decided to not proceed to second-stage surgery and continued his life with the spacer. Finally, a total of 24 patients were included while 20 (83.3%) patients had completed all questionnaires correctly and were included for final HRQoL analysis. A flowchart is given in Fig. 5.

## Microbiology

**Fig. 6:**
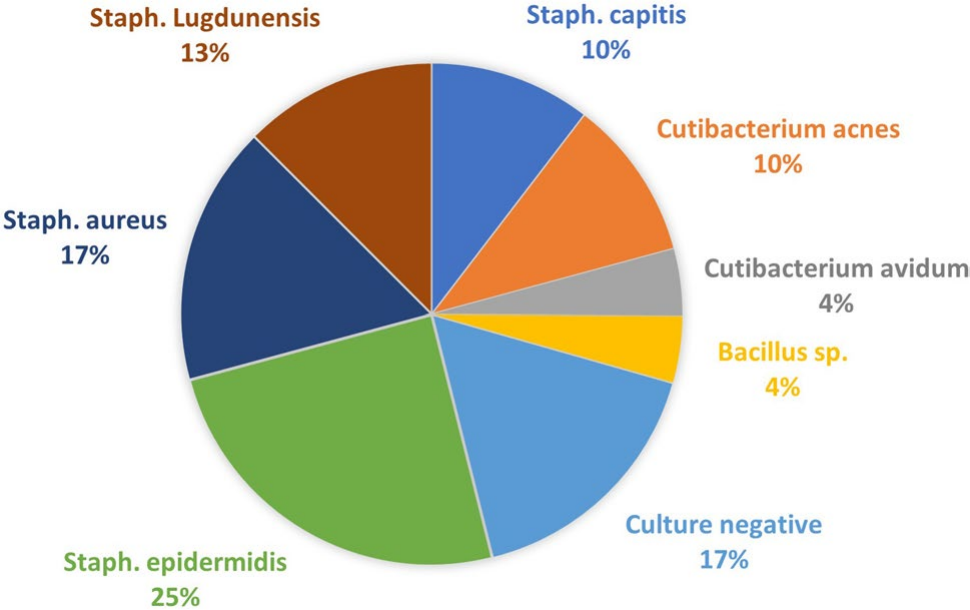
Microorganisms cultured at first-stage surgery

No microorganisms were found in 4 (16.7%) cases before or during first-stage surgery. In these cases, diagnosis was based on clinical presentation, laboratory work-up, including synovial analysis, and intraoperative findings. A total of 3 (12.5%) patients had positive microbiological results (*Staphylococcus epidermidis*) during second-stage surgery. Nevertheless, infection control was achieved in all 3 cases without requiring further surgery until latest follow-up. A summary of the microbiological results at first- and second-stage surgery is given in Table 1 and Fig. 6.

**Table 1:** Microorganisms cultured at first- and second-stage surgery

Causative microorganisms	First-stage	Second-stage
Culture negative	4 (16.7%)	21 (87.5%)
Single organisms	17 (70.8%)	3 (12.5%)
* Staph. epidermidis*	5 (20.8%)	3 (12.5%)
* Staph. capitis*	2 (8.3%)	
* Staph. lugdunensis*	3 (12.5%)	
* Staph. aureus*	4 (16.7%)	
* Cutibacterium acnes*	2 (8.3%)	
* Bacillus* sp.	1 (4.2%)	
Polymicrobial	3 (12.5%)	
* Cutibacterium acnes*	1 (4.2%)	
* Cutibacterium avidum*	2 (8.3%)	
* Staph. epidermidis*	2 (8.3%)	
* Staph. capitis*	1 (4.2%)	
Total	*24*	*24*

## Complications, infection control, and implant survivorship

The average age at first-stage surgery was 70 years (range 53.6–83.7; SD 9), and the mean American Society of Anesthesiologists Score (ASA) was 2.6 (range 2–3).

The overall mechanical spacer-related complication rate was 4.2% with one case of spacer dislocation which was treated with closed reduction. An additional round of spacer exchange had to be performed in 2 (8.3%) patients (three-stage revision surgery) for persistent wound drainage, before they proceeded to reimplantation. There were no other revisions or complications during the spacer period.

**Fig. 7:**
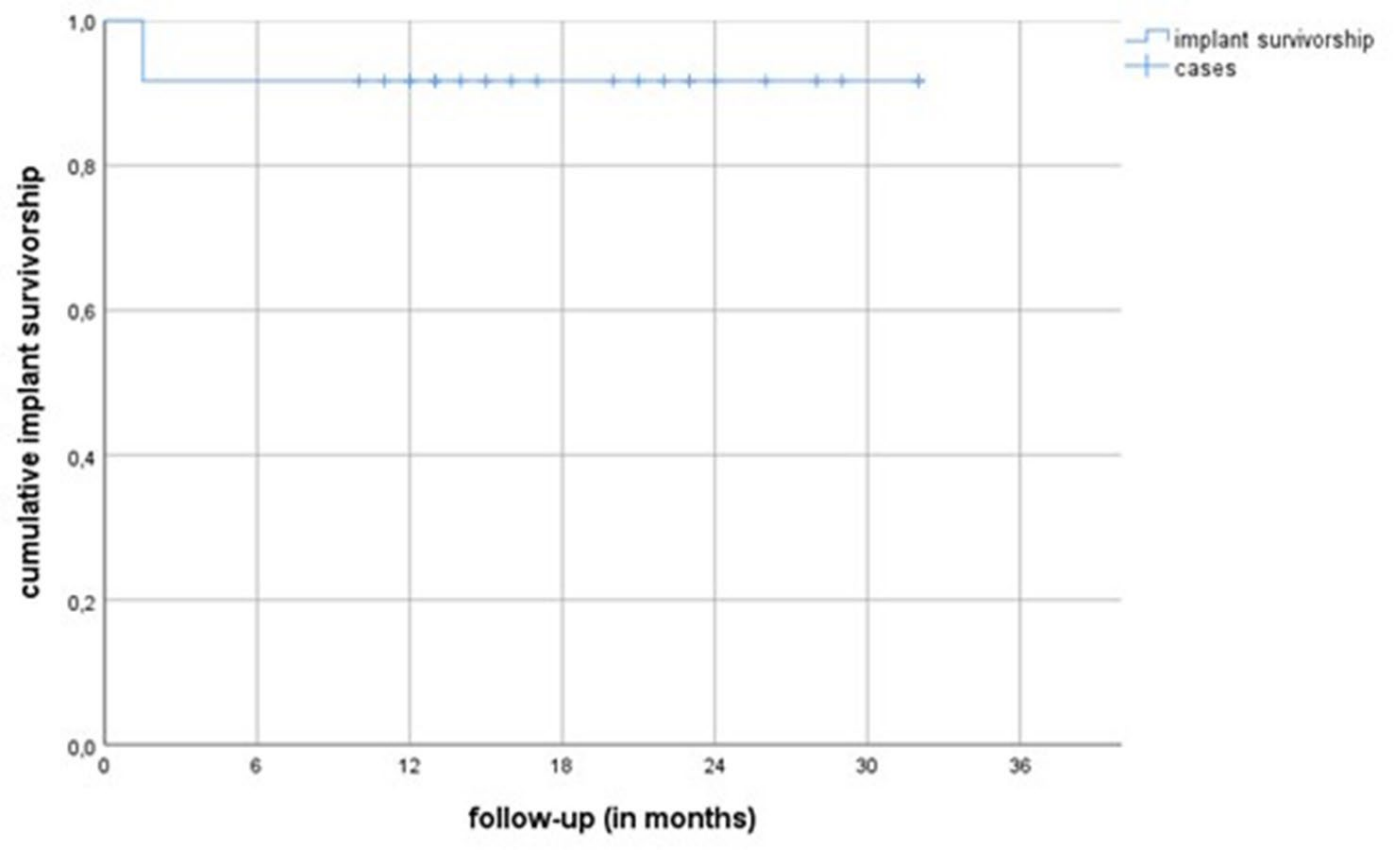
This graph shows the implant survivorship curve (Kaplan–Meier) with implant revision for any reason as its end point

We found an implant survivorship of 91.7% with implant revision for any reason as its endpoint at final follow-up. Figure 7 shows the implant survivorship curve (Kaplan–Meier) with implant revision for any reason as its endpoint. We had one case of early loosening of an uncemented femoral stem. Intraoperatively a persistent infection was evident and another round of two-stage revision was performed before infection control was finally achieved. Furthermore, we had one case of periprosthetic femur fracture after a low-impact fall, which was treated by open reduction and internal fixation and implant retention. Also, three cases of dislocation had occurred; two were successfully treated with closed reduction, while one patient underwent replacement of the acetabular cup due to recurrent dislocations. Finally, we had one case of postoperative peroneal nerve palsy, most likely due to traction trauma during second-stage surgery, which showed almost a full recovery (strength level of 4/5) at our latest follow-up. Table 2 summarizes all complications. The mean follow-up time was 19 (range 7–32, SD 7.2) months after reimplantation.

**Table 2:** Spacer-related complications and complications after reimplantation of new prosthesis

Complications	Spacer period		After reimplantation of new prosthesis	
	Occurrence	Requiring unplanned spacer revision	Occurrence	Requiring unplanned implant revision
Dislocation	1 (4.2%)	0	3 (12.5%)	1 (4.2%)
Periprosthetic fracture	0		1 (4.2%)	0
Implant revision for persistent wound drainage	2 (8.3%)	2 (8.3%)	0	
Recurrent infection	0		1 (4.2%)	1 (4.2%)
Nerve damage	0		1 (4.2%)	0
Total	3 (12.5%)	2 (8.3%)	6 (25%)	2 (8.3%)

The final reimplantation rate was 96% with a mean interim period of 90 days (median 84, range 17–253, SD 48.9). Infection control as defined by the Delphi criteria [33] was achieved in 23 out of 24 (95.8%) patients. One patient was diagnosed with recurrent PJI during re-revision surgery because of early loosening of the femoral stem (see above). Cement-free fixation at reimplantation was achieved in 14 (58.3%) patients. 6 (25%) patients received a “hybrid” fixation with cementation of the stem and a press-fit fixation of the acetabular cup. 4 (16.7%) patients received cementation of the stem and acetabular cup; a summary is given in Table 3. Full-weight bearing was allowed in 9 (37.5%) patients immediately after second-stage surgery; 15 (62.5%) patients were recommended to do partial-weight bearing for 6 weeks. At our latest follow-up, mean range-of-motion of the operated hip was 100/0/0° (range 80–130/0/0°) for hip flexion/extension.

**Table 3:** Fixation of implants at second-stage surgery

Type of implant and fixation	Femoral stem	Acetabular cup
Cementation	10 (41.7%)	4 (16.7%)
Press-fit/cement-free	14 (58.3%)	20 (83.3%)

## Patient-reported health-related quality of life (HRQoL)

**Fig. 8:**
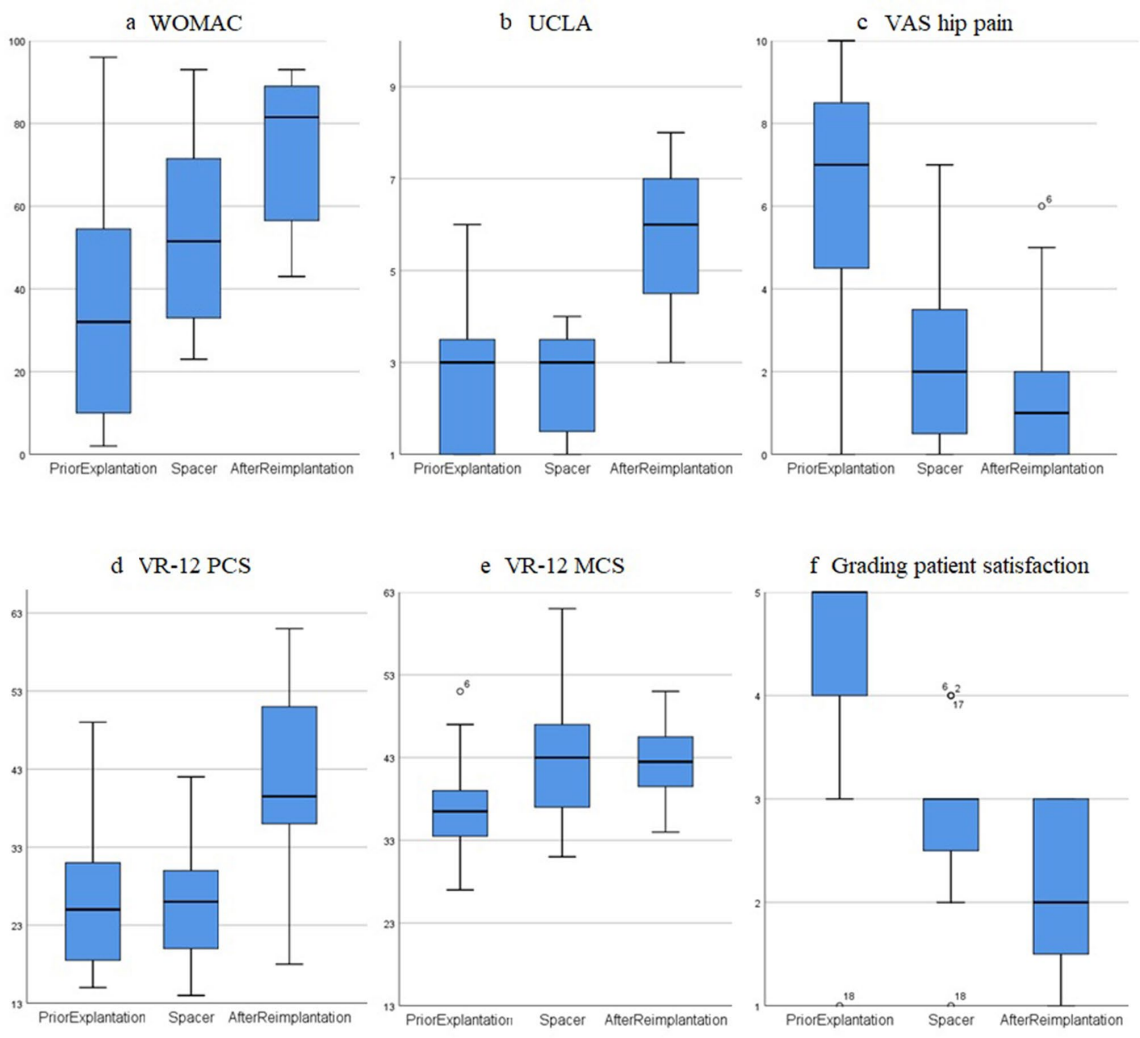
Box plots are presenting patient-reported health-related quality of life (HRQoL) scores for **a** WOMAC, **b** UCLA, **c** VAS for hip pain, **d** VR-12 PCS (physical component score), **e** VR-12 MCS (mental component score) and **f** grading for overall patient satisfaction for the three points in time (shortly prior to explantation, prior to reimplantation with spacer in situ, and after reimplantation of new prosthesis)

In Table 4 and Fig. 8, the HRQoL scores are summarized and visualized for the three points in time.

**Table 4:** Patient-reported health-related quality of life (HRQoL) scores shortly prior to explantation, during spacer period, and after reimplantation of new prosthesis

Questionnaire	1. Prior to explantation	2. Prior to reimplantation with spacer in-situ	3. After reimplantation at final follow-up
WOMAC	35 (SD 27.5)	54 (SD 22.9)	75* (SD 17.1)
UCLA	2.6 (SD 1.4)	2.6 (SD 1.1)	5.7* (SD 1.6)
VR-12 (PCS)	26 (SD 8.8)	26 (SD 7.7)	42* (SD 13)
VR-12 (MCS)	37 (SD 5.7)	43* (SD 7.3)	43 (* to 1.) (SD 4.9)
VAS hip pain	6.5 (SD 2.9)	2.3* (SD 2)	1.5 (* to 1.) (SD 1.8)
Grading patient satisfaction	4.4 (SD 1)	2.9* (SD 0.7)	2.1* (SD 0.8)

A pairwise analysis was conducted; the level of significance was set at *p* < 0.05 and is marked with * and written in bold letters. For better comparison with other studies, the WOMAC score was converted to a 100-point-scale with “0 being poor and 100 being the best possible outcome.” The Visual Analog Scale (VAS) for hip pain was recorded on a 0 to 10 scale with “0 = no pain” and “10 = worst pain”. The Grading for patient overall satisfaction was recorded on a 1 to 5 scale with “1 = extremely pleased, 2 = very pleased, 3 = pleased, 4 = disappointed, 5 = extremely disappointed”

The mean WOMAC score (for better comparison with other studies, the WOMAC score was converted to a 100-point-scale, 0 being poor and 100 being the best possible outcome) improved from 35 (range 2–96; SD 27.5) shortly prior to first-stage surgery, to 54 (range 24–93; SD 22.9) during the interim period (*p* = 0.12), to 75 (range 48–93; SD 17.1) after final reimplantation (*p* = 0.008).

The UCLA activity score did not change after first-stage surgery with a mean of 2.6 (range 1–6; SD 1.4) and 2.6 (range 1–4; SD 1.1), respectively. But it improved significantly to a mean of 5.7 (range 3–8; SD 1.6) (*p* = 0.001) after reimplantation of the new prosthesis.

VR-12 comprises two components, the physical component score (PCS-12) and the mental component score (MCS-12). The PCS-12 did not change after first-stage surgery with a mean of 26 (range 15–49; SD 8.8) and 26 (range 16–42; SD 7.7), respectively. But it improved significantly after reimplantation of the new prosthesis to a mean of 42 (range 18–61; SD 13) (*p* = 0.002). The MCS-12 improved significantly from 37 (range 27–51; SD 5.7) prior to explantation to 43 (range 31–61; SD 7.3) during the spacer period (*p* = 0.017), without a further improvement after second-stage surgery with 43 (range 34–51; SD 4.9).

The VAS for hip pain improved from a mean of 6.5 (range 0–10; SD 2.9) before explantation to 2.3 (range 0–7; SD 2) during the spacer period (*p* = 0.002), to 1.5 (range 0–6; SD 1.8) after reimplantation of the new prosthesis (*p* = 0.62).

The grading system (1 = extremely pleased, 2 = very pleased, 3 = pleased, 4 = disappointed, 5 = extremely disappointed) for overall patient satisfaction improved significantly from a mean of 4.4 (range 1–5; SD 1) prior to explantation to 2.9 (range 1–4; SD 0.7) during spacer period (p = 0.01) to 2.1 (range 1–3; SD 0.8) after reimplantation of the new prosthesis (*p* = 0.043).

## Discussion

**Fig. 9:**
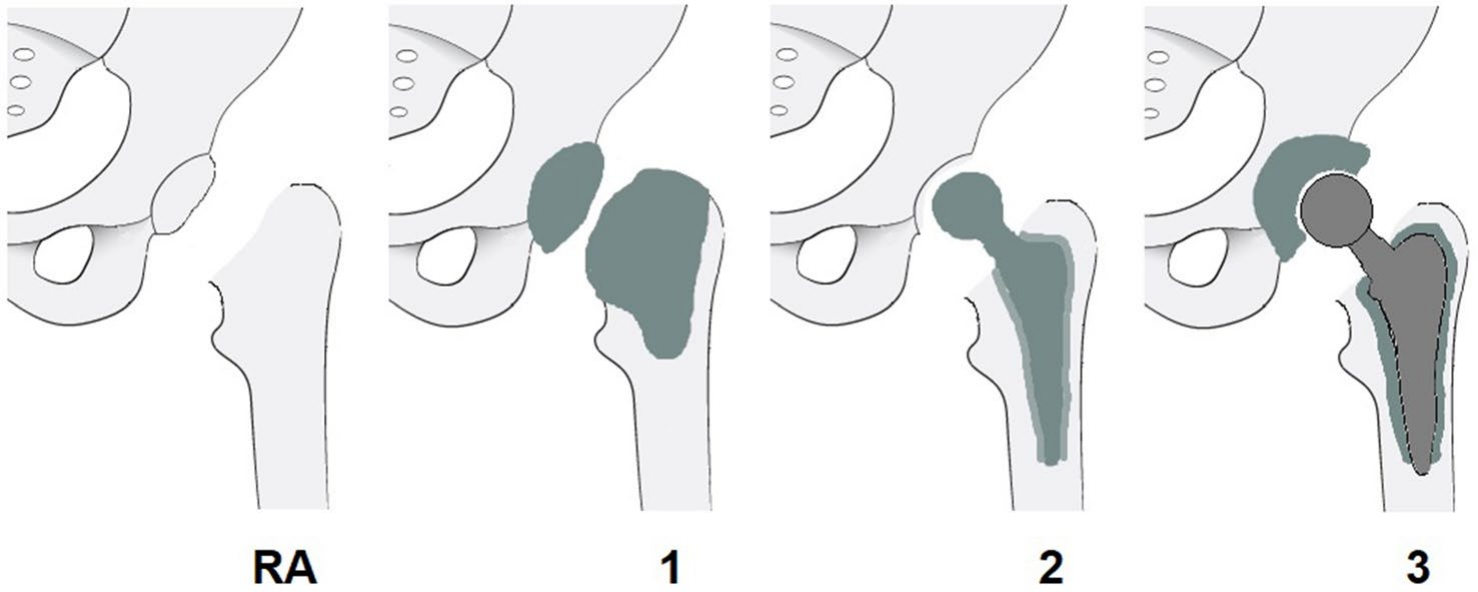
Introduction of a new hip spacer classification system (HSCS). Types 2 and 3 are mobile spacers and can be further categorized as either A = commercially available preformed components, B = commercially available molds, or C = custom-made

Two-stage revision remains the “gold standard” treatment for most chronically infected and complex total hip arthroplasty infections [10–14]. In the setting of two-stage revision a broad variety of different static and articulating spacers has been described in the literature [34]. To simplify comparison between different spacer designs, we suggest establishing a new “hip spacer classification system” (HSCS) and therefore have introduced our proposal in Table 5 and Fig. 9. This easy-to-comprehend classification system gives the reader an overview of the various spacer types and classifies them in four main categories. Its aim is to help the clinician to compare and discuss results of different spacer systems.

**Table 5:** Introduction of a new hip spacer classification system (HSCS)

Type		Explanation
RA	Resection arthroplasty	No articulation (Girdlestone hip)
1	Static spacer	PMMA cement cap implantation, either femoral or femoral + acetabular
2	Hemi-spacer	Comparable to a fixed-head hemiarthroplasty without implantation of an acetabular cap
3	Articulating spacer	Comparable to a total hip arthroplasty, articulation within the spacer

Types 2 and 3 are mobile spacers and can be further categorized as either A = commercially available preformed components, B = commercially available molds, or C = custom-made

## Infection control, complication rates, and implant survivorship

Yang et al. [28] described the results of 31 patients who were treated with a molded articulating PMMA hip spacer (HSCS 3B) and had an overall spacer complication rate of 64.4%. This high spacer complication rate makes molded articulating cement hip spacers an unpopular choice. The PROSTALAC system (Depuy, Warsaw, Indiana) [35] is a commercially available articulating spacer system (HSCS 3A) created from specialized metal and plastic components coated in antibiotic-loaded cement. The availability of this device is limited, the costs high, and at present it is not approved for use in the European Union. This prompted the development of various custom-made articulating spacers. Tsung et al. [36] published the results of 76 patients treated with the CUMARS technique (HSCS 3C), which is similar to the PROSTALAC system and consists of a regular polyethylene acetabular liner cemented into the acetabulum and a regular femoral stem fixed with antibiotic-loaded acrylic cement. The overall spacer complication rate was 22.4% with almost half of the complications being spacer dislocations. To further improve this technique and reduce the dislocation rate, Lausmann et al. [37] introduced the ENDO-spacer (HSCS 3C) using a dual mobility polyethylene liner instead of a regular polyethylene liner. The overall spacer complication rate was reduced to 16.7% with a total of 6.7% dislocations. Despite this improvement, our experience shows that there is no need of any polyethylene liner in the setting of a hip spacer as they did not provide lower dislocation rates compared to our polyethylene-free design (10.5% and 6.7% vs. 4.2%, respectively).

Faschingbauer et al. [38] and Jung et al. [39] published their results using a Hemi-Spacer design (HSCS 2B/2C and 2B, respectively) in 128 and 82 patients, respectively. The overall spacer complication rates were 19.6% and 48.8%, respectively. Infection control rates after reimplantation were not provided. Jones et al. [40] analyzed the influence of different Hemi-Spacer designs (HSCS 2 A/B/C) in a total of 155 patients on the rate of spacer complications (overall complication rate was 26%) and concluded that optimal restoration of offset and leg length and not a specific design is associated with mechanical complications such as dislocation or fracture. The Hemi-Spacer (HSCS 2) has the advantage of a less demanding surgical technique and potentially lower costs. However, the subjective pain level may be elevated because of the bone–cement or bone–metal articulation. Furthermore, its hemi-articulation leads to acetabular bone wear and therefore can result in increased acetabular bone stock loss.

Our custom-made articulating spacer design (HSCS 3C) allows for optimal reconstruction of the hip anatomy, including balanced leg length and optimal offset restoration, with a polyethylene-free design. In our cohort we only had one (4.2%) spacer dislocation that was successfully treated with closed reduction. The use of a “deliberately loosely cemented” stem reduces the risk for periprosthetic fracture or fracture of the spacer, while enabling an easy removal at second-stage surgery. In our series, we had no periprosthetic fractures or fractures of the spacer. Recently published prior studies [18, 35–41] showed significantly higher dislocation and fracture rates with 4–19% and 7–14%, respectively. A summary of recently published literature concerning spacer-related complications using mobile spacer designs is given in Table 6.

**Table 6:** Recently published literature concerning spacer-related complications using mobile spacers (HSCS 2 and 3)

Author	Year	HSCS	*N*	Spacer period	Overall complications	Revision	Dislocation	Fracture	Drainage/persistent infection
Matar [41]	2019	–	29	105 d	13.8%	–	3.4%	–	10.4%
Jones^a^ [40]	2019	2A/B/C	155	–	26%	–	9%	15%	4%
Jung [39]	2009	2B	82	90 d	49.8%	14.1%	17%	23.8%	9%
Fasching-bauer [38]	2015	2B/C	138	–	19.6%	13.2%	10.2%	9.4%	–
Yang [18]	2019	3B	31	86 d	64.4%	45%	19.4%	13%	32.2%
Lausmann [37]	2018	3C	30	54 d	16.7%	–	6.7%	3.4%	3.4%
Tsung [36]	2014	3C	76	143 d	22.4%	13.2%	10.5%	7.9%	–
Lunz^b^	2021	3C	24	90 d	12.5%	8.3%	4.2%	0%	8.3%

^a^Review, ^b^current study, d days, – no information provided, HSCS Hip Spacer Classification System (RA resection arthroplasty, 1 static spacer, 2 hemi-spacer, 3 articulating spacer; A preformed, B molded, C custom-made)

Infection control according to Delphi criteria [33] was achieved in 95.8% after a mean follow-up time of 19 (range 7–32, SD 7.2) months, which is superior to most reported rates from previous series [18, 35, 36, 39, 41]. Only one patient (4.2%) required another two-staged revision for persistent PJI. But our follow-up time was significantly shorter than in most other series. Nevertheless, persistent PJI is usually diagnosed during the first 12 months after surgery. In our opinion the mean follow-up time of 19 months is, therefore, sufficient to give an adequate short-time follow-up impression of the here described technique.

If no microorganisms were found before or during first-stage surgery, complication rates were 50% (2/4) in our cohort with one patient undergoing another course two-stage revision for persistent PJI and another patient undergoing an additional round of spacer exchange for persistent wound drainage. In our opinion, this underlines the importance of an adequate and accurate diagnostic workup to be able to perform a targeted antibiotic therapy after first-stage surgery.

In our cohort, implant survivorship was 91.7% after a mean follow-up time of 19 months. Aseptic revision rate after reimplantation was 4.2% with one case of recurrent dislocation that subsequently underwent exchange of the acetabular cup. We had no cases of aseptic loosening, but a further follow-up needs to confirm these preliminary short-term results, especially for the cemented implants. Recent studies by Chalmers et al. [35] and Tsung et al. [36] found aseptic loosening rates of 10% and 13% at 5 and 6.7 years, respectively. A summary of literature concerning infection control, implant survivorship, and complication rates after reimplantation of the new prosthesis is given in Table 7.

**Table 7:** Summary of recently published literature concerning infection control, implant survivorship, and complication rates in two-stage revision surgery using a mobile hip spacer design

Author	Year	HSCS	N	Follow-up	Infection control	Implant survivor-ship	Revision	Dislocation	Fracture	Aseptic loosening
Jones^a^ [40]	2019	2A/B/C	155	6.5 y	82%	73%	27%	7.1%	2%	3.9%
Chalmers [35]	2018	3C	131	5 y	88%	77%	23%	10%	2%	10%
Lunz^b^	2021	3C	24	1.5 y	95.8%	91.7%	8.3%	12.5%	4.2%	0%

^a^Review, ^b^current study, y = years, HSCS Hip Spacer Classification System with (RA resection arthroplasty, 1 static spacer, 2 hemi-spacer, 3 articulating spacer; A preformed, B molded, C custom-made)

## Patient-reported health-related quality of life (HRQoL) scores

HRQoL scores have not been studied as extensively as reinfection or complication rates in the setting of PJI; a summary of literature is given in Table 8. Poulsen et al. [42] analyzed in his cross-sectional study PROMs following two-staged revisions and reported significantly lower scores compared to the general population. In a systematic review by Rietbergen et al. [43], the average WOMAC score was 73 after two-stage revision of 185 patients with a mean follow-up of 70 months. This is consistent with our results, as our mean WOMAC score significantly improved from 35 to 75 (*p* = 0.001) and our mean UCLA score from 2.6 to 5.7 (*p* = 0.001), respectively. By taking the poor baseline situation, old age, and comorbidity into account, we think, that the achieved results are quite satisfactory.

**Table 8:** Summary of literature concerning HRQoL scores in the setting of two-stage revision for PJI

Author	Year	HSCS	N	Mean age	Prior explantation	With spacer	After reimplantation
Matar [41]	2019	–	29	63y	–	–	WOMAC 76.5
Yang [28]	2019	3B	31	56y	–	HHS 48.5	HHS 89.5
Lausmann [37]	2018	3C	30	70y	HHS 34	HHS 48	–
Chalmers [35]	2018	3C	131	65y	HHS 58	HHS 71	HHS 81
Rietbergen^a^ [43]	2016	–	154	65y	–	–	WOMAC 73 PCS 35, MCS 49
Lunz^b^	2021	3C	20	70y	WOMAC 35 PCS 26, MCS 37 UCLA 2.6	WOMAC 54 PCS 26, MCS 43 UCLA 2.6	WOMAC 75 PCS 42, MCS 43 UCLA 5.7

^a^Review, ^b^current study, y years, – no information provided, SF-12 and VR-12 are based on a PCS (physical component score) and a MCS (mental component score), HSCS Hip Spacer Classification System (RA resection arthroplasty, 1 static spacer, 2 hemi-spacer, 3 articulating spacer; A preformed, B molded, C custom-made)

We believe that it is important to underline that our patients did not lose their mobility after implantation of the spacer but gained significant pain relief already during the interim period. Both, the mean VAS for hip pain and mean grading score for patient satisfaction improved after first-stage surgery significantly to 2.3 from 6.5 and 2.9 from 4.4 (*p* = 0.001 in both cases), respectively.

## Custom-made articulating hip spacer technique

The spacer technique described here allows exact reconstruction of leg-length and offset due to the modular construction of the femoral stem and head. The metal-on-cement articulation prevents from acetabular bone wear and, therefore, preserves acetabular bone stock and simultaneously allows physiological joint motion, at least partial weight-bearing and early patient mobilization already during the interim period. Furthermore, we were able to show that our spacer design provides significant pain relief already during the interim period as well as high overall patient satisfaction. It can be easily adopted in other clinics. In our opinion, it is not mandatory to use exactly the same antibiotic-loaded bone cement (Palacos R + G cement, Heraeus, plus additional vancomycin powder) or femoral stem (Weber, Zimmer) or metal head component(DePuy Synthes). We just recommend using an appropriately sized metal head as well as an appropriately sized regular metal stem to achieve optimal reconstruction of the hip anatomy (especially balanced leg length and optimal offset). This provides sufficient stability and, therefore, prevention of spacer dislocation or fracture (only one dislocation and no spacer-related fractures had occurred in our cohort) and is at the same time cost efficient. Lausmann et al. [37] described a similar spacer technique with a polyethylene dual mobility liner to prevent spacer dislocation and reported a dislocation rate of 6.7%. We achieved comparable results (our spacer dislocation rate was 4.2%) without a costly polyethylene dual mobility liner, and therefore saving expenses and reducing the risk for biofilm formation. Furthermore, we have recently published that the here described custom-made hip spacer with a metal-on-cement articulation shows no relevant metallic wear [44]. To the best of our knowledge, the cement wear of any spacer was not yet quantified. But we assume that the cement wear of the spacer design described here should be lower than that of spacers with a cement–cement articulation, especially in knee spacers, which have a significantly bigger articulation surface. The metal-on-cement articulation prevents from acetabular bone wear and, therefore, preserves acetabular bone stock. In contrast to that, we believe a hemi-spacer design (HSCS 2) has an increased risk for acetabular erosions and hip pain. In our experience, only true articulating hip spacers (HSCS 3) allow pain-free physiological joint motion and early patient mobilization during the interim period.

## Limitations of this study

The retrospective nature of the study design with all its inherent limitations and the relatively small number of patients included in this study must be considered. This is a common problem because of the low rate of PJIs of 0.5–2% [1–3]. Therefore, even major joint arthroplasty centers do not take care of a lot of PJI cases yearly.

Furthermore, not all PJIs at our department were treated with the here-described articulating spacer technique in the given period (see flowchart in Fig. 5). Therefore, a selection bias could have happened towards healthier patients with good bone stock having been treated with the articulating spacer system described here.

Our reported incidence of recurrent or persistent PJI and aseptic loosening was lower than in most other published studies, as was our follow-up period after final reimplantation. It is probable that with a longer follow-up period higher rates of reinfection and aseptic loosening will be evident. This calls for a mid- to long-term follow-up of our cohort.

## Conclusions

We have introduced a custom-made articulating hip spacer technique that allows early patient mobilization with a good range of motion and significant pain relief already during the interim period. Complication rates during the spacer-period were low while overall satisfaction rates were high. After final reimplantation excellent rates for short-term implant survivorship and infection control were achieved. We believe this spacer technique can be easily adopted in other clinics with experience in revision arthroplasty to improve patient outcome in the demanding setting of chronic PJI.

## References

[1] Sukeik M, Haddad FS (2019). Periprosthetic joint infections after total hip replacement: an algorithmic approach. Sicot J.

[2] Phillips JE, Crane TP, Noy M, Elliott TS, Grimer RJ (2006). The incidence of deep prosthetic infections in a specialist orthopaedic hospital: a 15-year prospective survey. J Bone Jt Surg Br.

[3] Dale H, Fenstad AM, Hallan G, Havelin LI, Furnes O, Overgaard S, Pedersen AB, Kärrholm J, Garellick G, Pulkkinen P, Eskelinen A, Mäkelä K, Engesæter LB (2012). Increasing risk of prosthetic joint infection after total hip arthroplasty. Acta Orthop.

[4] Shohat N, Goswami K, Tan TL, Yayac M, Soriano A, Sousa R, Wouthuyzen-Bakker M, Parvizi J (2020). 2020 Frank Stinchfield Award: Identifying who will fail following irrigation and debridement for prosthetic joint infection. Bone Joint J.

[5] Fowler TJ, Sayers A, Whitehouse MR (2019). Two-stage revision surgery for periprosthetic joint infection following total hip arthroplasty. Ann Transl Med.

[6] Winkler T, Trampuz A, Hardt S, Janz V, Kleber C, Perka C (2014). Periprosthetic infection after hip arthroplasty. Orthopade.

[7] Nguyen M, Sukeik M, Zahar A, Nizam I, Haddad FS (2016). One-stage exchange arthroplasty for periprosthetic hip and knee joint infections. Open Orthop J.

[8] Pangaud C, Ollivier M, Argenson JN (2019). Outcome of single-stage versus two-stage exchange for revision knee arthroplasty for chronic periprosthetic infection. EFORT Open Rev.

[9] Svensson K, Rolfson O, Kärrholm J, Mohaddes M (2019). Similar risk of re-revision in patients after one- or two-stage surgical revision of infected total hip arthroplasty: an analysis of revisions in the Swedish Hip Arthroplasty Register 1979–2015. J Clin Med.

[10] Sukeik M, Haddad FS (2009). Two-stage procedure in the treatment of late chronic hip infections–spacer implantation. Int J Med Sci.

[11] Dombrowski ME, Wilson AE, Wawrose RA, O'Malley MJ, Urish KL, Klatt BA (2020). A low percentage of patients satisfy typical indications for single-stage exchange arthroplasty for chronic periprosthetic joint infection. Clin Orthop Relat Res.

[12] Palmer JR, Pannu TS, Villa JM, Manrique J, Riesgo AM, Higuera CA (2020). The treatment of periprosthetic joint infection: safety and efficacy of two stage versus one stage exchange arthroplasty. Expert Rev Med Devices.

[13] Kildow BJ, Della-Valle CJ, Springer BD (2020). Single vs 2-stage revision for the treatment of periprosthetic joint infection. J Arthroplasty.

[14] Anagnostakos K, Fink B (2018). Antibiotic-loaded cement spacers—lessons learned from the past 20 years. Expert Rev Med Devices.

[15] Charette RS, Melnic CM (2018). Two-stage revision arthroplasty for the treatment of prosthetic joint infection. Curr Rev Musculoskelet Med.

[16] Durbhakula SM, Czajka J, Fuchs MD, Uhl RL (2004). Spacer endoprosthesis for the treatment of infected total hip arthroplasty. J Arthroplasty.

[17] Shahpari O, Mousavian A, Elahpour N, Malahias MA, Ebrahimzadeh MH, Moradi A (2020). The use of antibiotic impregnated cement spacers in the treatment of infected total joint replacement: challenges and achievements. Arch Bone Jt Surg.

[18] Yang FS, Lu YD, Wu CT, Blevins K, Lee MS, Kuo FC (2019). Mechanical failure of articulating polymethylmethacrylate (PMMA) spacers in two-stage revision hip arthroplasty: the risk factors and the impact on interim function. BMC Musculoskelet Disord.

[19] Pitto RP, Sedel L (2016). Periprosthetic joint infection in hip arthroplasty: is there an association between infection and bearing surface type?. Clin Orthop Relat Res.

[20] Lass R, Giurea A, Kubista B, Hirschl AM, Graninger W, Presterl E, Windhager R, Holinka J (2014). Bacterial adherence to different components of total hip prosthesis in patients with prosthetic joint infection. Int Orthop.

[21] Workgroup Convened by the Musculoskeletal Infection Society (2011). New definition for periprosthetic joint infection. J Arthroplasty.

[22] Parvizi J, Gehrke T, Chen AF (2013). Proceedings of the international consensus on periprosthetic joint infection. Bone Jt J.

[23] Izakovicova P, Borens O, Trampuz A (2019). Periprosthetic joint infection: current concepts and outlook. EFORT Open Rev.

[24] Preininger B, Janz V, von Roth P, Trampuz A, Perka CF, Pfitzner T (2017). Inadequacy of joint aspiration for detection of persistent periprosthetic infection during two-stage septic revision knee surgery. Orthopedics.

[25] Muhlhofer HML, Knebel C, Pohlig F, Feihl S, Harrasser N, Schauwecker J, von Eisenhart-Rothe R (2018). Synovial aspiration and serological testing in two-stage revision arthroplasty for prosthetic joint infection: evaluation before reconstruction with a mean follow-up of twenty seven months. Int Orthop.

[26] Tan TL, Kheir MM, Rondon AJ, Parvizi J, George J, Higuera CA, Shohat N, Chen AF (2018). Determining the role and duration of the "antibiotic holiday" period in periprosthetic joint infection. J Arthroplasty.

[27] Becker A, Kreitmann L, Triffaut-Fillit C, Valour F, Mabrut E, Forestier E, Lesens O, Cazorla C, Descamps S, Boyer B, Chidiac C, Lustig S, Montbarbon E, Batailler C, Ferry T (2020). Duration of rifampin therapy is a key determinant of improved outcomes in early-onset acute prosthetic joint infection due to Staphylococcus treated with a debridement, antibiotics and implant retention (DAIR): a retrospective multicenter study in France. J Bone Jt Infect.

[28] Yang J, Parvizi J, Hansen EN, Culvern CN, Segreti JC, Tan T, Hartman CW, Sporer SM, Della Valle CJ (2020). Mark Coventry Award: microorganism-directed oral antibiotics reduce the rate of failure due to further infection after two-stage revision hip or knee arthroplasty for chronic infection: a multicentre randomized controlled trial at a minimum of two years. Bone Jt J.

[29] Ware J, Kosinski M, Keller SD (1996). A 12-Item Short-Form Health Survey: construction of scales and preliminary tests of reliability and validity. Med Care.

[30] Bellamy N, Buchanan WW, Goldsmith CH, Campbell J, Stitt LW (1833). Validation study of WOMAC: a health status instrument for measuring clinically important patient relevant outcomes to antirheumatic drug therapy in patients with osteoarthritis of the hip or knee. J Rheumatol.

[31] Zahiri CA, Schmalzried TP, Szuszczewicz ES, Amstutz HC (1998). Assessing activity in joint replacement patients. J Arthroplasty.

[32] Williamson A, Hoggart B (2005). Pain: a review of three commonly used pain rating scales. J Clin Nurs.

[33] Diaz-Ledezma C, Higuera CA, Parvizi J (2013). Success after treatment of periprosthetic joint infection: a Delphi-based international multidisciplinary consensus. Clin Orthop Relat Res.

[34] Abdel MP, Barreira P, Battenberg A, Berry DJ, Blevins K, Font-Vizcarra L, Frommelt L, Goswami K, Greiner J, Janz V, Kendoff DO, Limberg AK, Manrique J, Moretti B, Murylev V, O’Byrne J, Petrie MJ, Porteous A, Saleri S, Sandiford NA, Sharma V, Shubnyakov I, Sporer S, Squire MW, Stockley I, Tibbo ME, Turgeon T, Varshneya A, Wellman S, Zahar A (2019). Hip and knee section, treatment, two-stage exchange spacer-related: proceedings of international consensus on orthopedic infections. J Arthroplasty.

[35] Chalmers BP, Mabry TM, Abdel MP, Berry DJ, Hanssen AD, Perry KI (2018). Two-stage revision total hip arthroplasty with a specific articulating antibiotic spacer design: reliable periprosthetic joint infection eradication and functional improvement. J Arthroplasty.

[36] Tsung JD, Rohrsheim JA, Whitehouse SL, Wilson MJ, Howell JR (1813). Management of periprosthetic joint infection after total hip arthroplasty using a custom made articulating spacer (CUMARS); the Exeter experience. J Arthroplasty.

[37] Lausmann C, Citak M, Hessling U, Wolff M, Gehrke T, Suero EM, Zahar A (2018). Preliminary results of a novel spacer technique in the management of septic revision hip arthroplasty. Arch Orthop Trauma Surg.

[38] Faschingbauer M, Reichel H, Bieger R, Kappe T (2015). Mechanical complications with one hundred and thirty eight (antibiotic-laden) cement spacers in the treatment of periprosthetic infection after total hip arthroplasty. Int Orthop.

[39] Jung J, Schmid NV, Kelm J, Schmitt E, Anagnostakos K (2009). Complications after spacer implantation in the treatment of hip joint infections. Int J Med Sci.

[40] Jones CW, Selemon N, Nocon A, Bostrom M, Westrich G, Sculco PK (2019). The influence of spacer design on the rate of complications in two-stage revision hip arthroplasty. J Arthroplasty.

[41] Matar HE, Stritch P, Emms N (2019). Two-stage revisions of infected hip replacements: subspecialisation and patient-reported outcome measures. J Orthop.

[42] Poulsen NR, Mechlenburg I, Søballe K, Lange J (2018). Patient-reported quality of life and hip function after 2-stage revision of chronic periprosthetic hip joint infection: a cross-sectional study. Hip Int.

[43] Rietbergen L, Kuiper JW, Walgrave S, Hak L, Colen S (2016). Quality of life after staged revision for infected total hip arthroplasty: a systematic review. Hip Int.

[44] Lunz A, Sonntag R, Kretzer JP, Jaeger S, Bormann T, Streit MR, Beckmann NA, Lehner B, Omlor GW (2020). Hip spacers with a metal-on-cement articulation did not show significant surface alterations of the metal femoral head in two-stage revision for periprosthetic joint infection. Materials (Basel).

